# Comparative Effects of Intravaginal Progesterone Sponge Durations (Short vs. Long Duration) With or Without Probiotics on Vaginal Microbiota Balance and Reproductive Performance in Black Iraqi Goats

**DOI:** 10.1155/vmi/2031122

**Published:** 2026-08-10

**Authors:** Mohammedali J. Ghafil, Mustafa A. K. Al-Taie, Ansam K. Mohammed

**Affiliations:** ^1^ Department of Anatomy and Histology, College of Veterinary Medicine, University of Wasit, Wasit, Iraq, uowasit.edu.iq; ^2^ Department of Biology, College of Science, University of Baghdad, Baghdad, Iraq, uobaghdad.edu.iq; ^3^ Department of Microbiology, College of Veterinary Medicine, University of Baghdad, Baghdad, Iraq, uobaghdad.edu.iq

**Keywords:** early pregnancy, goats, *Lactobacillus* spp., progesterone sponge, vaginal discharge

## Abstract

Intravaginal progesterone sponges have been widely utilized to synchronize estrus in goats; however, extended use may be accompanied by vaginal microbial imbalance, vaginal discharge abnormalities, and diminished reproductive performance. Intravaginal probiotics have been suggested as an alternative to antibiotics for addressing vaginal dysbiosis, but their effects across varying synchronization durations have not been thoroughly studied in goats. The current study was conducted to assess the effect of intravaginal probiotic use in association with various progesterone sponge durations on vaginal microbiota and reproductive efficiency in Black Iraqi goats. Forty‐four adult goats (2–5 years) were randomly assigned to four groups (*n* = 11) for 7 and 14 days of treatment with a progesterone sponge alone or in combination with probiotics. Two strains of *Lactobacillus* (*L. plantarum* and *L. acidophilus*) were used in a lyophilized product (2 × 10^9^ CFU/sponge). The following were assessed: cultivable vaginal bacteria (total bacteria, *Enterobacteriaceae*, *Lactobacillus*), score of vaginal discharge, onset of estrus and duration of estrus, and early pregnancy rate. Larger *Enterobacteriaceae* and total bacterial counts, and lower *Lactobacillus* counts, were observed with prolonged retention (14 days) compared to the short‐duration protocol (*p* < 0.05). Probiotic treatment showed significantly higher *Lactobacillus* counts (2.75 ± 0.69 vs. 0.44 ± 0.25 log CFU/mL) (*p* < 0.001) and significantly lower *Enterobacteriaceae* and total counts, especially in the long protocol (*Enterobacteriaceae* 4.37 ± 0.36 vs. 5.20 ± 0.44; total 5.26 ± 0.43 vs. 6.14 ± 0.44 log CFU/mL), with a significantly earlier onset of estrus (29.82 ± 0.66 vs. 33.00 ± 1.18 h), longer duration of estrus (29.91 ± 0.79 vs. 24.73 ± 0.84 h), and milder discharge (Fisher’s exact test, *p* = 0.01–0.03) than controls. The early pregnancy rate was numerically higher in the probiotic‐treated groups but not statistically significant (*p* > 0.05). Overall, the duration of sponge use was associated with vaginal health and reproductive responses, with short‐sponge duration protocols with high‐dose *Lactobacillus* associated with decreased dysbiosis and enhanced vaginal health, suggesting a non‐antibiotic strategy that warrants testing in larger studies for its potential role in conception.

## 1. Introduction

Black Iraqi goats are dairy animals with significant economic value in Iraq. To enhance their reproductive efficiency, effective management of reproductive practices is a primary concern. Among the numerous factors controlling reproductive success is the technique used to synchronize estrus for artificial insemination (AI). In goats, estrus synchronization can be managed by different protocols, such as using multiple or single hormones. One of the most prevalent techniques for estrous synchronization is the use of progesterone‐impregnated intravaginal sponges, which enhance regular estrous cycles [1]. However, these protocols might produce problems in the vagina, such as abnormal vaginal discharge, vaginitis, and irritation, especially if the sponges remain for a long time [2, 3]. Many authors reported that extended retention of these intravaginal sponges may alter the microbiota of the vagina. As progesterone has immunosuppressive properties, it could facilitate the proliferation of opportunistic pathogenic bacteria, which may cause dysbiosis of the vagina and raise the possibility of ascending reproductive tract infection, which may demand the use of antibiotics [4, 5].

In small ruminants, several studies indicated that removal of vaginal sponges was associated with abnormal vaginal discharges, unpleasant vaginal smell, and localized inflammatory reaction, which was associated with reduced fertility rate [6]. Therapeutic techniques, such as antibiotics, used to control vaginal opportunistic bacteria in small ruminants can affect the natural microbiota and increase antimicrobial resistance among these microorganisms [7, 8]. The presence of drug residues in milk and meat poses health risks; therefore, one must be careful when using antibiotics and seek alternative methods for managing health and reproduction in goats [6]. Many studies have investigated the effects of probiotics on human vaginal health, indicating that *Lactobacillus* enhances the vaginal mucosal barrier and competitively inhibits the colonization of opportunistic bacteria by producing substances like bacteriocin and hydrogen peroxide (H_2_O_2_), which suppress the growth of these bacteria and help maintain a low pH [9, 10].

Recently, in animal studies, probiotics have been suggested as an alternative to antibiotics, thereby restoring the healthy vaginal microbiota and improving fertility [11, 12]. Despite these positive outcomes in ovine studies, evidence from caprine studies remains limited. We recently reported that intravaginal probiotics can affect the microbiota of the vagina and decrease the incidence of vaginitis in Black Iraqi goats synchronized with progesterone sponges; this effect was comparable to enrofloxacin [13]. The effect of sponge duration on the probiotic response, however, has not been studied in goats. Since caprine and ovine immune responses and vaginal microbiota differ, it should be studied whether caprine can benefit from vaginal probiotics in the same way. The objective of this study was to conduct research into the influence of *Lactobacillus* spp. (*L. plantarum* and *L. acidophilus*) on vaginal microbial populations, estrus duration, vaginal secretions, and early pregnancy rate in black Iraqi goats that had been synchronized with progesterone‐impregnated sponges. We hypothesized that reducing the sponge retention period and combining it with high‐dose intravaginal probiotics would reduce vaginal dysbiosis. The primary endpoints were the cultivable vaginal bacteria (*Enterobacteriaceae*, *Lactobacillus*, and total bacteria) at the time of sponge removal; the secondary endpoints were the vaginal discharge score, estrus onset and duration, and early pregnancy rate.

## 2. Materials and Methods

The study was carried out on a commercial farm in Baghdad, Iraq, during the breeding season. Forty‐four adult black Iraqi goats, aged 2–5 years, were randomly divided into four separate groups, each group (*n* = 11). Prior to the application of synchronization protocols, all animals underwent vaginal examinations to ensure normal reproductive health. For estrus synchronization, intravaginal sponges infused with 40 mg of fluorogestone acetate (FGA) (Pharmplex, Australia) were used for each group. All goats were healthy, multiparous, nonpregnant, and cyclic by pretreatment. The animals were maintained under uniform housing and nutrition throughout the study. Eight mature bucks of proven fertility (two per group; buck‐to‐doe ratio 2:11) were used for natural mating and were distributed equally across all groups to control for the male effect.

The four experimental groups included the first group, which was treated with the FGA sponge only for 7 days. The second group was treated with FGA sponge combined with probiotics for 7 days. The third group was treated with the FGA sponge only for 14 days. The fourth group was treated with FGA sponge combined with probiotics for 14 days. The sponges were removed at the same time in all groups, so that the goats in the short treatment program received the sponges 7 days before the planned removal date; the goats in the long treatment program received the sponges for 14 days prior to removal. During sponge removal, all animals were injected with 5 mg of prostaglandin f2α.

Two probiotic strains, *Lactobacillus plantarum* and *Lactobacillus acidophilus*, are contained in a commercially lyophilized probiotic product “Vitalactic B” (Vitane Pharma GmbH, Germany) at a concentration of 2 × 10^9^ CFU per capsule used for probiotic preparation. The contents of the capsule were carefully opened, placed in a sterile 2‐mL Eppendorf tube, and suspended in 1.5 mL of de Man, Rogosa, and Sharpe (MRS) broth (Oxoid, UK). Then, the mixture was injected into the vaginal sponge using a sterile 21‐G syringe (3 mL). Lyophilized *Lactobacillus* spp. in the “Vitalactic B” product do not require prior cultivation and activation, as they rapidly resume metabolic activity after rehydration [14]. To prevent solution overflow, all dosages were injected into six distinct places beneath the sponge once it was placed on the sponge applicator.

At the time of sponge withdrawal, the vaginal discharge characteristics, such as amount and presence of hemorrhagic or purulent secretions, were assessed and scored as 0 = no discharge or negligible, 1 = clear but detectable discharge, and 2 = abundant with hemorrhagic or purulent discharge [15].

For colony‐forming unit (CFU) counts (log CFU/mL), vaginal swabs were collected from each group both before and shortly after the sponge was removed using sterile plastic stick swabs, which were gently rotated across the vaginal mucosa. The swab was placed in 9 mL of maximum recovery diluent (MRD; HiMedia, India) at neutral pH. The samples were refrigerated until processing, which took approximately 4 h.

Tubes were vortexed for 60 s to suspend the bacteria, serial dilutions (10^−1^–10^−6^) were prepared in MRD, then 0.1 mL from each selected dilution was spread in: (a) sheep blood agar (Oxoid, UK) for counting of total bacteria, (b) MacConkey agar (Oxoid, UK) for *Enterobacteriaceae* counts, and (c) MRS agar (Oxoid, UK) for *Lactobacillus* count. The plates have been incubated at 37°C for 24–48 h under either aerobic or microaerophilic conditions, as required. Plates that contain 30–300 colonies were used for counting, and the results were recorded as log10 CFUs per milliliter (CFU/mL).

Estrus was observed three times daily (08:00 a.m., 03:00 p.m., and 08:00 p.m.) following sponge removal. The parameters were observed for 5 days: estrus onset, estrus behavior, and duration of estrus. According to Solihati et al. [1], the estrus behavior was rated on a four‐point scale. Score zero (0) indicated absence of estrus; score (1) represented silent heat; score two (2) reflected restlessness, characterized by intense bleating, self‐rubbing against surfaces, tail wagging while attention to the male; score three (3) signified standing heat, accompanied by heightened restlessness, a higher degree of bleating, self‐rubbing against surfaces, tail wagging while attention to the male, and reduced appetite.

Vaginal cytology was used to assess estrus duration, based on the presence of superficial and keratinized cells, which were observed daily for 5 days following sponge removal. The dominance of superficial cells duration indicates estrus phase.

For early pregnancy evaluation, one day after the sponge was removed, bucks with crayon marks were introduced for natural mating at a 2:11 ratio (bucks:does). Mating behavior is observed at the same time as estrus detection, and crayon marks on the does indicate successful mounting. Pregnancy diagnosis was performed by using a portable veterinary ultrasonography (DP‐10, Mindray, China), 28 ± 3 days after mating.

Data analysis was carried out using SAS software (version 9.4; SAS Institute Inc., Cary, NC, USA) [16]. Normality was tested using the Shapiro–Wilk test. Continuous data (bacterial count, onset, duration of estrus) are presented as means and SDs and compared by independent‐samples *t*‐tests (two‐sided). Vaginal discharge scores and early pregnancy rates were categorical and analyzed as frequencies and percentages, and tested using Fisher’s exact test. A *p* value < 0.05 was considered significant. For each comparison, a priori and pairwise: probiotic vs control in each protocol, and 7 vs 14 days protocol in each treatment. No factorial (group × time) interaction model was used, nor was there a multiplicity adjustment for the analysis of continuous outcomes (which were analyzed using pairwise *t*‐test); as such, results are interpreted as planned pairwise contrasts.

### 2.1. Ethical Approval

This study was approved by the Animal Care and Use Committee of the College of Veterinary Medicine, University of Baghdad, Iraq, under license number P.G./2735 on 2/11/2025.

## 3. Results

### 3.1. *Enterobacteriaceae* Counts

Before sponge insertion (Day 0), the number of vaginal *Enterobacteriaceae* did not differ between either group within either protocol (*p* > 0.05); thus, the baseline bacterial load was similar in the two groups (Table 1). A significant increase in the number of *Enterobacteriaceae* occurs after sponge removal across all experimental groups, but the increase was less pronounced in the probiotic‐treated groups than in the controls. No statistically significant differences were observed between the control and probiotic groups after removal (*p* > 0.05); under the short protocol (7 days), counts increased from 2.29 ± 0.28 log CFU/mL to 4.12 ± 0.78 log CFU/mL in the control group and from 2.14 ± 0.30 log CFU/mL to 3.43 ± 0.77 log CFU/mL in the probiotic group. Under the long protocol (14 days), bacterial growth was more pronounced in the probiotics‐treated group, with a significantly lower population of 4.37 ± 0.36 log CFU/mL than in the control group (5.20 ± 0.44 log CFU/mL; *p* < 0.05). Furthermore, the number of *Enterobacteriaceae* in each group was significantly higher with the long protocol (14 day) compared to the short protocol (7 day) (control: 4.12 ± 0.78 vs. 5.20 ± 0.44 log CFU/mL; *p* = 0.001; probiotic: 3.43 ± 0.77 vs. 4.37 ± 0.36 log CFU/mL; *p* = 0.002).

**TABLE 1:** *Enterobacteriaceae* count (log CFU/mL) in control and probiotic‐treated goats in short (7 day) and long (14 day) protocols.

Day	Control	Probiotics	*p* (treatment)
0 days	2.29 ± 0.28	2.14 ± 0.30	0.24
7 days	4.12 ± 0.78	3.43 ± 0.77	0.05
0 days	2.15 ± 0.31	2.22 ± 0.43	0.67
14 days	5.20 ± 0.44	4.37 ± 0.36	< 0.001
*p* (duration: 7 vs 14)	0.001	0.002	—

*Note:* Data are given as mean ± SD (*n* = 11 per group), *p* (Treatment) = control vs. probiotic, *p* (duration) = 7‐day vs 14‐day protocol, independent‐samples *t*‐test (two‐sided), *p* < 0.05 was considered significant.

### 3.2. *Lactobacillus* Counts

Before sponge insertion (Day 0), there was no significant difference between the control and probiotic groups for either protocol (*p* > 0.05), indicating similar starting populations (Table 2). After the removal of sponges, however, divergent microbiological trends developed. The population of *Lactobacillus* in the control groups, both under the short (7 day) and long (14 day) protocol, showed a significant reduction from 1.04 ± 0.45 to 0.44 ± 0.25 log CFU/mL and from 1.48 ± 0.61 to 0.65 ± 0.24 log CFU/mL, respectively, with no statistically significant difference between the two intervention period lengths (*p* > 0.05). On the other hand, the intravaginal route of probiotic administration induced a strong increase in *Lactobacillus* counts (1.13 ± 0.41 to 2.75 ± 0.69 log CFU/mL in the short protocol and 1.18 ± 0.31 to 1.92 ± 0.12 log CFU/mL in the long protocol), and the short protocol resulted in significantly higher bacterial load than the long protocol (*p* < 0.05). Accordingly, there was a significant increase in *Lactobacillus* counts in the probiotic groups compared with the corresponding controls for both protocols (*p* < 0.001); the absolute maximum counts were observed in the probiotic group of the short protocol.

**TABLE 2:** *Lactobacillus* count (log CFU/mL) in control and probiotic‐treated goats in short (7 day) and long (14 day) protocols.

Day	Control	Probiotics	*p* (treatment)
0 days	1.04 ± 0.45	1.13 ± 0.41	0.63
7 days	0.44 ± 0.25	2.75 ± 0.69	< 0.001
0 days	1.48 ± 0.61	1.18 ± 0.31	0.17
14 days	0.65 ± 0.24	1.92 ± 0.12	< 0.001
*p* (duration: 7 vs 14)	0.06 (NS)	0.003	—

*Note:* Data are given as mean ± SD (*n* = 11 per group); *p* (treatment) = control vs. probiotic; *p* (duration) = 7‐day vs, 14‐day protocol, independent‐samples *t*‐test (two‐sided), *p* < 0.05 was considered significant.

### 3.3. Total Bacterial Count

Total bacterial counts were similar between the control and probiotic groups in each protocol prior to sponge insertion (Day 0; *p* > 0.05) (Table 3). After sponge removal, total bacterial counts increased significantly across all experimental groups; however, the increase was smaller in the probiotic‐treated groups than in their control counterparts. The short (7‐day) protocol showed counts of 3.60 ± 0.39 log CFU/mL in the control group and 3.49 ± 0.39 log CFU/mL in the probiotic group before the removal and of 5.22 ± 0.64 log CFU/mL in the control group and 4.84 ± 0.60 log CFU/mL in the probiotic group after the removal; no significant difference between the two groups was observed after removal (*p* > 0.05). However, microbial proliferation was more evident under the long protocol (14 days), and the probiotic group had a significantly lower total bacterial load than the control (5.26 ± 0.43 vs. 6.14 ± 0.44 log CFU/mL, *p* < 0.001). As for the protocol length, the total counts of bacteria in the control groups were significantly different under the two protocols (6.14 ± 0.44 log CFU/mL for the long protocol and 5.22 ± 0.64 log CFU/mL for the short protocol; *p* < 0.05), but no significant differences were found between the two protocols in the probiotic‐treated groups (5.26 ± 0.43 log CFU/mL for the long protocol and 4.84 ± 0.60 log CFU/mL for the short protocol; *p* > 0.05).

**TABLE 3:** Total bacterial count (log CFU/mL) in control and probiotic‐treated goats in short (7 day) and long (14 day) protocols.

Day	Control	Probiotics	*p* (treatment)
0 days	3.60 ± 0.39	3.49 ± 0.39	0.52
7 days	5.22 ± 0.64	4.84 ± 0.60	0.17
0 days	3.73 ± 0.17	3.56 ± 0.40	0.22
14 days	6.14 ± 0.44	5.26 ± 0.43	< 0.001
*p* (duration: 7 vs 14)	0.001	0.08 (NS)	—

*Note:* Data are given as mean ± SD (*n* = 11 per group), *p* (treatment) = control vs. probiotic; *p* (duration) = 7‐day vs, 14‐day protocol; independent‐samples *t*‐test (two‐sided), *p* < 0.05 was considered significant.

### 3.4. Vaginal Discharge Score

The distribution of the vaginal discharge scores at the time of sponge removal revealed significant differences between the probiotic‐treated and control groups using both synchronization protocols (Table 4).

**TABLE 4:** Vaginal discharge scores at time of sponge removal in control and probiotic‐treated goats in short (7 day) and long (14 day) protocols.

Score	Control		Probiotics	
	7 days control	7 days probiotics	14 days control	14 days probiotics
0	0 (0/11)	0 (0/11)	0 (0/11)	0 (0/11)
1	45.5 (5/11)	100 (11/11)	18.2 (2/11)	72.7 (8/11)
2	54.5 (6/11)	0 (0/11)	81.8 (9/11)	27.3 (3/11)
*p* (treatment: control vs. probiotic)	0.01		0.03	
*p* (duration: 7 day vs. 14 day)	Control: 0.36 (NS)		Probiotic: 0.21 (NS)	

*Note:* Data are % (*n*/11), *p* (treatment) = control vs. probiotic, *p* (duration) = 7‐day vs. 14‐day protocol within groups, Fisher’s exact test was used to analyze the data, with *p* < 0.05 considered significant.

Abbreviation: NS, not significant.

In the short protocol (7 day), all the goats treated with the probiotics presented mild, clear discharge (Score 1: 11/11 [100%]). However, the control group had a higher proportion of purulent/hemorrhagic discharge (6/11, 54.5%) and a lower proportion of clear discharge (5/11, 45.5%), which showed a significant difference between the two groups (Fisher’s exact test, *p* = 0.01).

Similarly, during the long (14 day) protocol, purulent or hemorrhagic discharge was more common in the control group (9/11, 81.8%, score 2). In contrast, most of the goats that received the probiotic had a clear discharge (8/11 [72.7%] and 3/11 [27.3%] had scores of 1 and 2, respectively), resulting in a statistically significant difference (Fisher’s exact test, *p* = 0.03).

### 3.5. The Estrus Onset

Probiotics had a significant effect on estrus advancement under both synchronization protocols (Table 5). In the 7‐day protocol, probiotic‐treated goats entered estrus significantly earlier than controls (29.82 ± 0.66 vs. 33.00 ± 1.18 h; *p* < 0.001), and the same pattern was observed in the 14‐day protocol (35.27 ± 3.80 vs. 40.36 ± 4.55 h; *p* = 0.01). Protocol duration also showed significant differences in the interval to estrus, with a shorter interval after the short protocol than after the long protocol in both the control and probiotic groups (*p* < 0.001 for both).

**TABLE 5:** Estrus onset (h) in control and probiotic‐treated goats in short (7 day) and long (14 day) protocols.

Group	Control	Probiotics	*p* (treatment)
7 days	33.00 ± 1.18	29.82 ± 0.66	< 0.001
14 days	40.36 ± 4.55	35.27 ± 3.80	0.01
*p* (duration: Day 7 vs Day 14)	< 0.001	< 0.001	—

*Note:* Data are given as mean ± SD (*n* = 11 per group), *p* (treatment) = control vs. probiotic; *p* (duration) = 7‐day vs. 14‐day protocol, independent‐samples *t*‐test (two‐sided), *p* < 0.05 was considered significant.

### 3.6. Estrus Duration

The duration of estrus was significantly affected by both the synchronization protocol used and probiotic treatment, as described in Table 6. Probiotic administration consistently prolonged estrus, as evidenced by higher values in the treated groups compared to controls under both the 7‐day (29.91 ± 0.79 vs. 24.73 ± 0.84 h; *p* < 0.001) and 14‐day protocols (24.27 ± 2.68 vs. 20.37 ± 2.30 h; *p* = 0.002). Protocol length was also important; in both groups, the short (7 day) protocol resulted in a significantly longer estrus period compared to the long (14 day) protocol (*p* < 0.001).

**TABLE 6:** Estrus duration (h) in control and probiotic‐treated goats in short (7 day) and long (14 day) protocols.

Group	Control	Probiotics	*p* (treatment)
7 days	24.73 ± 0.84	29.91 ± 0.79	< 0.001
14 days	20.37 ± 2.30	24.27 ± 2.68	0.002
*p* (duration: Day 7 vs Day 14)	< 0.001	< 0.001	—

*Note:* Data are given as mean ± SD (*n* = 11 per group), *p* (treatment) = control vs. probiotic, *p* (duration) = 7‐day vs. 14‐day protocol, independent‐samples *t*‐test (two‐sided), *p* < 0.05 was considered significant.

### 3.7. Early Pregnancy Rate

The results in Table 7 indicate that no significant difference was observed in early pregnancy rate between probiotic‐treated groups and controls, although the values were numerically higher in the probiotic‐treated groups (probiotic short vs. control: 81.8% (9/11) vs. 63.6% (7/11), *p* = 0.64; probiotic long vs. control: 63.6% (7/11) vs. 45.5% (5/11), *p* = 0.67). Similarly, there was no significant difference between the two groups in the length of the synchronization protocol (63.6% vs. 45.5%, *p* = 0.67; 81.8% vs. 63.6%, *p* = 0.64, in the control and probiotic groups, respectively). The higher early pregnancy rate in the probiotic treatment group should be viewed only as a numerical trend, given the small group sizes (*n* = 11).

**TABLE 7:** Early pregnancy rate % in control and probiotic‐treated goats in short (7 day) and long (14 day) protocols.

Protocol	Control	Probiotics	*p* (treatment)
7 days	7/11 (63.6%)	9/11 (81.8%)	0.64 (NS)
14 days	5/11 (45.5%)	7/11 (63.6%)	0.67 (NS)
*p* (duration: Day 7 vs Day 14)	0.67 (NS)	0.64 (NS)	—

*Note:* Data are *n*/11 (%),  *p* (treatment): probiotic compared to control within each protocol; *p* (duration): 7‐day protocol compared to 14‐day protocol within each treatment, all comparisons were made using Fisher’s exact test, and *p* < 0.05 was considered significant. There were no significant differences in the comparisons.

Abbreviation: NS, not significant.

## 4. Discussion

The present study revealed that the intravaginal retention period of progesterone‐releasing sponges is associated with the composition of cultivable vaginal bacteria, which, in turn, is associated with reproductive responses in black Iraqi goats. In particular, intravaginal administration of *Lactobacillus* spp. has been shown to alleviate microbial dysbiosis resulting from prolonged sponge retention. The longer (14 day) synchronization protocol showed a marked microecological change, including increased Gram‐negative opportunists (particularly within the *Enterobacteriaceae* family) and an increase in the total number of culturable bacteria, whereas the shorter (7 day) synchronization protocol resulted in a more balanced cultivable profile and better estrus response. In both no‐probiotic (control) groups, this change was accompanied by a reduction in indigenous Lactobacilli and a higher proportion of mucopurulent discharge; however, the between‐duration difference in discharge was not statistically significant. Overall, these results indicate that it may be the local vaginal microenvironment of the insert, rather than progesterone exposure alone that is responsible for the changes in the vaginal microbial and inflammatory environment following withdrawal.

The negative effects of prolonged progestagen exposure on the following reproductive competence appear consistent with several previous investigations. In particular, Dogan et al. [17] found similar estrous response rates and conception rates in goats with either short‐ or long‐duration protocols. On the other hand, Thammasiri et al. [18] showed that although 14‐day treatment had no effect on circulating progesterone levels, it was associated with decreased both oocyte quality and early embryonic development. In line with this, Martínez‐Ros et al. [15] correlated a 14‐day insert retention period with reduced oocyte competency and embryonic development, and described a positive association between 7 days of in situ insert and increased follicular competency. The physiological observations are supplemented in our culture‐based microbiological work, which showed that as sponges were retained, there was a significant increase in *Enterobacteriaceae* and total bacterial counts, coupled with a significant decrease in *Lactobacillus* populations. Thus, it is proposed that the local microecological changes of the vagina due to sponge insertion and retention are yet another factor related to lower reproductive competence.

The experimental design of the present study included two different retention periods, unlike previous studies investigating only one retention period, such as Çortu and Şababoğlu Baytaroğlu [19], which associated progesterone sponge with dysbiosis in the vagina and purulent discharge, but did not pay attention to the time. In our data, we believe the length of sponge retention is a factor that influences vaginal microbiota balance and a relation with the variation in vagina’s discharge and early reproductive reactions. This agrees with the findings of Al‐Zubaidi et al. [20], who found that the 14‐day retention period caused severe vaginitis in small ruminants and affected the vagina’s ecosystem, which led to a reduction in the conception rate.

In the present study, the effectiveness of intravaginal administration of *Lactobacillus* in different synchronization intervals in goats was assessed as one of the main aims, as there is scarce information on the use of probiotic‐loaded sponges in goats (species in which most of the literature is restricted to ewes). The probiotic load (2 × 10^9^ CFU/sponge) was selected wisely, as previous studies with lower numbers were found to be less effective. In particular, Quereda et al. [11] used a 14‐day period of *Lactobacillus* spp. infusion at a lower dose (3 × 10^6^ CFU) and reported its safety profile, but did not see any significant clinical or bacteriological reduction in the incidence of clinical vaginitis and total bacterial loads. Similarly, *L. plantarum* at 1 × 10^8^ CFU/sponge or its cell‐free supernatant did not significantly reduce vaginitis or odor scores after 14 days, and there was no difference in pregnancy rates compared with the control groups [21]. These results are consistent with those of Guner et al. [12], who also reported a slight improvement in therapeutic efficacy with the cell‐free supernatant of *L. plantarum*. All of these data together show a “dose‐dependent and formulation‐dependent” effect. Therefore, it is possible that the 20 times higher bacterial load used in our current regimen explains the strong suppression of Gram‐negative opportunists, the decrease in bacterial counts, and the stimulation of cultivable lactic acid bacteria counts, especially in the prolonged protocol, which produced the most dysbiosis. Similarly, we have found in our previous study with black Iraqi goats that the same high dose of *L. plantarum* and *L. acidophilus* (2 × 10^9^ CFU/sponge) resulted in a significant decrease in the number of *Escherichia coli* and lower incidence of vaginitis among goats compared with the untreated groups [13].

The methodological and species‐specific differences might also explain the apparent rapidity of the probiotic effect observed here. Toquet et al. [6] performed metagenomic sequencing in ewes and found that intravaginal high‐dose treatment led to progressive modulation of the vaginal microbiota, with minimal modification at sponge removal and greater taxonomic modification during follow‐up sampling phases. In contrast, the culture‐based quantification was accompanied by an immediate change in the counts of cultivable *Lactobacillus*, *Enterobacteriaceae*, and total bacteria, especially at the time of device removal in the goat. However, these findings need to be interpreted with caution as they may be due to differences in the analytical method used (culture‐based versus NGS), the specific strains of *Lactobacillus* employed, the time these inserts were left in place, and host physiology, due to the different composition of the caprine and ovine vaginal ecosystem and localized immune response.

Vaginal discharge scores, along with clinical observations, were concurrent with the level of vaginal dysbiosis at the time of the removal of the sponges. Actually, a positive correlation existed between the occurrence of purulent/hemorrhagic discharge (Score 2) and higher *Enterobacteriaceae* loads, and higher total bacterial counts, particularly under the long‐term protocol. A high prevalence of mucopurulent or sanguineous exudate was observed, similar to the high prevalence reported by Penna et al. [22] after synchronization with progestin‐impregnated sponges. The probiotics‐treated groups, however, had less discharge, indicating a lower pathogenic load and helping maintain the *Lactobacillus* count to some extent. These observations confirm earlier research showing that estrus synchronization to optimize the vaginal environment can reduce vaginitis in the absence of antibiotic treatment [23]. Nonetheless, the type of progestagen and the properties of the intravaginal device can affect vaginal health and probiotic results. The application of *Lactobacillus plantarum* was beneficial in reducing purulent/hemorrhagic discharge in progestagen‐treated ewes FGA, but not in ewes treated with medroxyprogesterone acetate (MPA), and pregnancy rates were higher for progestagen type (FGA) than for the probiotic treatment [24]. The discharge and reproductive responses were assessed only in relation to FGA and cannot be applied to MPA‐based protocols; the progestagen–probiotic interaction needs to be explored in caprine models.

Both the length of the synchronization protocol and probiotic supplementation significantly affected the onset and length of estrus. The onset of estrus tended to be earlier in caprines receiving intravaginal *Lactobacillus*, regardless of the protocol used; however, the shortest interval to onset was noticed under the short protocol, regardless of treatment. This indicates that prolonged sponge retention is associated with a delayed onset of estrus, whereas probiotic supplementation was associated with an earlier onset, possibly through an improved post‐withdrawal vaginal environment. In contrast to Omontese et al. [25], who accelerated the onset of estrus hormonally by using chorionic gonadotropin (eCG), our results suggest a nonhormonal route through improved vaginal health. In addition, the estrus period was extended in the probiotic‐treated goats while it was shortened in the long‐term protocol. The duration of estrus in females, as observed in these dynamics, is in accordance with the study conducted by Salama et al. [26], which revealed that a short 7‐day FGA protocol improved the estrous response compared to a 14‐day FGA protocol in ewes, and Solihati et al. [1], who reported some differences in estrus duration dependent on the protocol used. The prolonged estrus period observed in the treated groups may reflect an improved vaginal environment after sponge removal, but estrous expression is a multifaceted phenomenon and cannot be attributed to a single factor in the experimental design.

There was a numerical increase in the early pregnancy rate in both probiotic‐treated groups, but this was not statistically significant (*p* > 0.05). Given the small sample size (*n* = 11), this difference should be interpreted with caution and regarded as a preliminary observation rather than definitive evidence of treatment efficacy. Given this conservative interpretation, the results are consistent with Guner and Guner [24], who reported an association of pregnancy outcomes with progestagen regimens more than with *Lactobacillus* supplementation, and with Guner et al. [12], who reported similar rates of conception in probiotic‐treated versus control ewes. On the contrary, the improved microbiological results and reduced abnormal vaginal discharge observed herein suggest improved vaginal condition during estrus synchronization with intravaginal *Lactobacillus*. Its direct effects on fertility, however, are speculative and should be tested in well‐powered, large‐scale clinical trials. The fact that the direction of the results is similar to that of Shallal et al. [23], who reported improved reproductive performance after intravaginal probiotic treatment in ewes, is reassuring, but the evidence in caprine models remains preliminary.

A number of constraints need to be recognized. The size of the experimental groups was small (11 animals each), and no power calculation was done beforehand, so the results on reproductive outcomes are exploratory. The pregnancy status was determined by transrectal ultrasound scanning at 28 ± 3 days and should be interpreted as the early pregnancy rate, as kidding rate, litter size, and late pregnancy loss were not monitored. Since the microbiological data are obtained by culturing bacterial populations, the full complex of nonculturable bacteria present in the vaginal community is not described, and the term “microbiota” here is used in a limited sense (a culturable subset). As far as possible, potential confounding factors such as parity, body condition score, cyclic status at the beginning of treatment, and the male effect were standardized but not included in the statistical model. Lastly, just a single progestagen (FGA) and sponge type were employed. Such constraints argue for a cautious interpretation of the current results and outline potential avenues for future research (larger, sequencing‐based, and well‐powered studies).

In conclusion, the present study suggests that the duration of sponge retention in the vagina is associated with the vaginal microecological balance and reproductive responses in black Iraqi goats. Prolonged retention was found to be associated with vaginal dysbiosis and severe vaginal discharge. A higher dose of intravaginal *Lactobacillus* spp. during estrus synchronization was associated with a more favorable profile of cultivable bacteria and less severe discharge. The early pregnancy rate did not differ significantly between the probiotic‐treated and control groups, but intravaginal *Lactobacillus* may be a novel, nonantibiotic approach to improving vaginal health during synchronization. However, its final effects on conception rates still need to be confirmed in large‐scale, higher‐powered clinical trials [26].

## Author Contributions

Mohammedali J. Ghafil and Mustafa A. K. Al‐Taie performed the practical field work and supervised the experimental procedures at the study farm.

Ansam K. Mohammed conducted the bacterial isolation and laboratory analyses.

Mohammedali J. Ghafil contributed to the data interpretation, manuscript writing, and final editing.

## Funding

This research did not obtain any specific financing from public, commercial, or nonprofit entities.

## Disclosure

All authors read and approved the final version of the manuscript.

## Conflicts of Interest

The authors declare no conflicts of interest.

## Data Availability

The data that support the findings of this study are available from the corresponding author upon reasonable request.
